# Access to denture restoration services under removable dentures subsidy program for adults aged 65 years and older in Taiwan- an interpretive approach

**DOI:** 10.1186/s12913-022-07504-6

**Published:** 2022-01-20

**Authors:** Kuan-Yu Chu

**Affiliations:** 1 Department of Stomatology, Taipei City Hospital Renai Branch, 10629 No. 10, Section 4, Renai Road, Daan District Taipei City, Taiwan; 2 University of Taipei, Taipei City, Taiwan

**Keywords:** Access, Removable dentures, Government subsidy, Geriatric dentistry

## Abstract

**Background:**

Access is an important issue in health equality. Availability of dental services and cost subsidies is an important factor affecting access to denture restoration for the elderly. This study aims to explore access to denture restoration services in the elderly removable denture’s subsidy program of  Taiwan.

**Methods:**

Access to the elderly removable dentures subsidy program was measured from two aspects, that is, availability of subsidies and payment for these services and the characteristics of patients and their treatment needs. The first aspect included reimbursements and the number and location of subsidy clinics, and the second aspect included the age and gender distribution of patients and denture types. Information on reimbursement regulations and the number and location of dental clinics providing subsidized services were obtained from the website of the Taoyuan City Public Health Bureau, Taoyuan Hospital, Department of Statistics and Ministry of Health and Welfare. Data on patient characteristics and denture type were obtained through a retrospective survey. We selected individuals who participated in the elderly removable denture’s subsidy program from 2015 to 2018 at the Geriatric Dentistry Department of Taoyuan Hospital. We conducted data analysis using an interpretive approach.

**Results:**

This study found that reimbursement amounts are inadequate, and the availability of subsidized services is low. Moreover, the proportion of male applications is slightly higher than that of females. In addition, among the applicants, removable partial dentures for single or two arches are the most common.

**Conclusions:**

Problems of insufficient numbers of contracted hospitals and low reimbursement amounts are observed in the subsidy program, which are the key factors affecting access to denture restoration services among the elderly. Policymakers should exclude wealthy individuals and offer subsidy only to low-income elderly individuals with missing teeth who are in dire need of financial support to improve their dental health.

**Supplementary Information:**

The online version contains supplementary material available at 10.1186/s12913-022-07504-6.

## Background

Access is an important issue in health equality and defined as the degree of fitness between patients and providers [1]. Since 1974, numerous scholars have proposed specific dimensions for measuring access, that is, the three dimensions of system and patient characteristics and access indicators. In addition, the measurement indices of access include availability, accessibility, acceptability, affordability, and adequacy [1–4]. Saurman later extended this theoretical framework to include awareness [5].

Population aging is increasing in developed and developing countries [6]. Concerns about population aging stem mainly from older people who require increased medical services [7]. Economic analyses focused on the financial impact of aging, which is expected to increase the burden on health and welfare services [8]. Specifically, low dental rehabilitation rates in an aging population mean that elderly individuals will require dental services [9]. Severe tooth loss and edentulism in older individuals are very common [10]. Among the elderly, systemic pathologies that reduce motor skills are common. Therefore, multidisciplinary management of the elderly should include oral care and rehabilitation to improve their quality of life [11]. Moreover, the nutrition status and quality of life of older adults are adversely affected when their missing teeth are not replaced [12, 13].

Removable dentures are an economical and affordable option for older individuals with missing teeth [14]. However, financial considerations may hinder older individuals from seeking medical treatment and obtaining dentures, thereby creating a gap in equitable medical treatment [15]. In Europe and the United States, the availability of dental services and price subsidies for dental treatments are important factors that affect access to dental care services for older adults [16]. In Europe and the United States, the availability of dental services and cost subsidies for dental treatment is an important factor affecting access to dental care services for older adults [17]. Evidence shows that older individuals are willing to avail dental services if government subsidies are available [16, 18, 19].

According to the 2012 National Health Interview Survey of the United States, 17.5% of individuals aged 65–74 years had missing teeth. This rate was 25.8% for individuals over the age of 75 years [20]. Taiwan’s oral condition survey in 2016 indicated that the average number of natural teeth in individuals between the ages of 65 and 74 years was 20.82. In this age group, the proportion of edentulous men was 35.6% and that of edentulous women was 32.4%. Among individuals over the age of 75 years, the average number of natural teeth was 16.71. Moreover, in this age group, 45.2% of men were edentulous, and 54.4% of women were edentulous [21]. In Taiwan, the prevalence of missing teeth is higher among women than among men, which was similarly observed in a US study [17]. Tsai (2003) pointed out that more than half of the population over the age of 65 years in Taiwan require new dental prostheses [22].

Taiwan became an aged society in 2018, and at the end of December 2019, the proportion of the population over the age of 65 years was 15.28% or approximately 3.6 million. However, the municipality of Taoyuan is relatively young, with only 12.11% of the population (approximately 272,000 individuals) over the age of 65 years [23]. Taoyuan is one of the few cities in Taiwan that provides a subsidy to non-low-income elderly individuals for removable dentures and thus is a suitable research setting. This study aims to explore access to dental restoration services in the elderly removable dentures subsidy program and establish an evaluation model of this medical policy to provide decision makers with a reference for improvement.

## Method and materials

This study focuses on the gap in access to denture restoration services and uses Penchansky and Thomas’s modified theory of access as the study design [1]. We measured access to the elderly removable dentures subsidy program from two aspects, that is, availability of subsidies and payment for these services and the characteristics of patients and their treatment needs. The first aspect included reimbursements and the number and location of subsidy clinics, and the second aspect included the age and gender distribution of patients and denture types.

Information on reimbursement regulations and the number and location of dental clinics providing subsidized services were obtained from the website of the Taoyuan City Public Health Bureau, Taoyuan Hospital, Department of Statistics and Ministry of Health and Welfare [24, 25].

To obtain information on patient characteristics and the denture type, we retrospectively investigated the individuals who participated in the elderly removable denture’s subsidy program for the period of 2015–2018 at the Geriatric Dentistry Department of Taoyuan Hospital. The individuals who participated in this program underwent an oral examination, and those who met the requirements were given removable dentures. The requirements for the participants were as follows: at least 65 years old with more than four missing teeth in a single jaw or more than two missing teeth with a free-end saddle, not including the third molar. In this survey, the following inclusion criteria were used: 1) a geriatric dentistry patient at Taoyuan Hospital during the period of 2015–2018; 2) completed the application procedure to participate in the removable dentures subsidy program for the elderly, specifically, underwent an oral examination, accomplished the application form and paid the application fee; and 3) completed the process, from the fabrication to the delivery of the removable dentures. The exclusion criteria included the following: 1) subsidy recipients under the age of 65 years, 2) applicants who failed to complete the delivery step by 31 December 2018 and 3) self-financed cases. Each subsidy applicant was required to complete the following steps: undergo an oral examination, accomplish the application form and submit the application form to the Municipal Public Health Bureau. All the participants provided informed consent. After the application process, the removable dentures were fabricated and delivered to the applicants, who were required to complete a satisfaction survey. The applicant’s name, gender, age, denture type, application fee amount and removable denture design were the most important items on the application form. In the present study, we obtained the oral examinations and application forms with information on denture type, application fee amount and design of the removable dentures. Dental assistants were responsible for administrative duties and taking photographs of participants with preoperative and postoperative dentures.

For the statistical analysis, IBM SPSS version 21 was used (IBM Corp., Armonk, NY, USA). Data analysis was conducted using an interpretive approach, as is customary in policy analysis. The Taipei City Hospital Research Ethics Committee approved the study protocol. (Grant numbers TCHIRB-11002013-E).

## Results

The single-jaw removable partial dentures (RPDs) subsidy is NT 15,000 (approximately USD 500). An RPD with fewer than three remaining teeth per jaw and the single-jaw complete dentures subsidy are NT 20,000 (approximately USD 667).

At the end of 2017, Taoyuan City had 13 administrative districts, and 151 dental clinics were contracted to provide subsidized services (availability) in the 13 administrative districts (accessibility). At the time, Taoyuan City had a total of 488 dental clinics (including hospitals).

In the retrospective survey, 432 applications were received during the study period, excluding five patients under the age of 65 years and four who failed to complete the entire process by the end of 2018. The individuals who obtained removable dentures at their own expense are excluded from the study. Thus, a total of 423 eligible individuals are included. Among them, 54.4% are male, and 45.6% are female, and the mean age is 76.3 years. Tables 1 and 2 shows that overall, the applications for both-jaw dentures (64.5%) outnumber those for single-jaw prostheses. In terms of the age group, the 65–74 years group (42.8%) is the largest, followed by the 75–84 years group (38.5%). The survey also shows that among those who applied for a single-jaw dentures subsidy, those who applied for RPDs account for the majority (64%), particularly upper-jaw RPDs (approximately one-third). Among the participants who applied for a both-arch dentures subsidy, the majority applied for both-arch full-mouth dentures and both-arch RPDs, which are both more than 20%.

**Table 1:** Characteristics who participated elderly removable dentures subsidy program in Geriatrics Dentistry of Taoyuan Hospital, 2015–2018, *n* = 423

	Age	Gender		Total	*p*- value†
		Female	Male		
Applied Single Arch	65–74	39	30	69	0.003
	75–84	30	31	61	
	85–94	4	15	19	
	95	0	1	1	
	Total	73	77	150	
Applied Both Arches	65–74	50	62	112	< 0.001
	75–84	56	46	102	
	85–94	14	45	59	
	Total	120	153	273	
Total	65–74	80	92	181	
	75–84	86	77	163	
	85–94	18	60	78	
	95	0	1	1	
	Total	193	230	423	

^†^ Pearson's Chi-square Test for Independence

**Table 2:** Denture types who applied removable dentures subsidy in Geriatrics Dentistry of Taoyuan Hospital, 2015–2018, *n* = 423

	Type	Times	Percent (%)
Applied Single Arch *n* = 150	LC	15	10.0
	LR	46	30.7
	LH	6	4.0
	UC	17	11.3
	UR	50	33.3
	UH	16	10.7
	Total	150	100
Applied Both Arches *n* = 273	BC	64	23.4
	BR	65	23.8
	BH	16	5.9
	UCLR	26	9.5
	UCLH	20	7.3
	URLC	20	7.3
	URLH	19	7.0
	UHLC	20	7.3
	UHLR	23	8.4
	Total	273	100

*U* Upper arch, *L* Lower arch, *C* Complete denture, *R* RPD (Removable Partial Denture), *H* RPD with remaining teeth≦3, *B* Both arches

## Discussions

This study measures the reimbursement of services, location and number of clinics providing subsidized services, the demographic characteristics of the participants and the denture type to evaluate access to removable dentures for the elderly. This study finds that a reimbursement of NT 15,000 is provided for RPDs, which is equivalent to 37.5% of the self-financed price in the hospital. Moreover, a reimbursement of NT 20,000 is given for complete or half dentures (remaining teeth ≦3), which is equivalent to 40% of the self-financed price in the hospital. From this point of view, the reimbursement system is inadequate. Among the 13 administrative regions of Taoyuan City, at least one contracted institution in each administrative region provides subsidized services and demonstrates satisfactory accessibility. Among the 488 dental clinics in the city, only 151 are willing to sign a subsidy contract, and availability is approximately 30%, which can be regarded as average. From the above two points, cost reimbursements and availability of dental services are important factors affecting access to denture restoration. These findings appear to be generally compatible with the results of previous studies [16].

The survey results show that the proportion of male applications is slightly higher than that of females, which is different in the general population of Taiwan, where older women have more missing teeth than older men [21]. The reason for this finding may be that men with missing teeth are relatively young and can easily go to the hospital to apply for a removable dentures subsidy. These results may also be a consequence of inadequate information on the subsidy provided to the public or insufficient medical institutions participating in the subsidy program.

In terms of the denture type, RPDs are the most common among the single-arch applicants, and RPDs and full dentures are the most common among the both-arch applicants. Some patients chose acrylic dentures, for which the reimbursement amount is the same as that for RPDs with a metal frame. High satisfaction is reported for both types of dentures, as reported in previous studies [26, 27]. Given the restrictions on the types of subsidized dentures, some patients experienced dry mouth or severe atrophy in the alveolar bone of their lower jaw. They complained that the complete denture retention was insufficient but lacked financial resources to obtain implant-supported removable dentures [28–30].

The study aims to provide suggestions for this subsidy policy and establish an evaluation model for similar policies. The findings of this study can help policymakers improve the removable dentures subsidy program for older adults. The Taiwanese government implemented the subsidy program for older individuals with missing teeth to help solve the problem of accessibility. However, provisions to limit access to the subsidy to applicants who are financially advantaged are necessary [31]. In this way, reimbursement amounts can be increased, which may improve dentists’ willingness to participate in the subsidy program and solve the problem of low availability.

This study has several limitations. The first limitation concerns the sample used in the study. This study uses the elderly dentures subsidy program of Taoyuan City as the research object. Nevertheless, a no exclusion clause in elderly dentures subsidy systems is rare. The generalizability of the results to populations with different socioeconomic backgrounds may be limited. Additionally, in terms of financial support, the government has a budget, and the elderly can ignore affordability. The satisfaction survey after the delivery of the dentures was conducted in an office setting, and reliability may not be high. Therefore, this study does not discuss acceptability. Future research could include the use of a verified structural satisfaction questionnaire for follow-up after the delivery of dentures to supplement the acceptability dimension of access to denture restoration services. Owing to the reasons, this study does not discuss acceptability and awareness as elements of access. Extensive research across the country is necessary to provide further evidence for policy improvement.

## Conclusion

Although the Taiwanese government provides a subsidy to older residents to help them overcome financial barriers to access denture restoration, the problems of insufficient numbers of contracted hospitals and low reimbursement amounts remain in the subsidy program. The two factors affect the elderly’s access to denture restoration. However, the available subsidy amount is low. Therefore, the willingness of dental professionals to participate in the subsidy program is also low, which may affect the availability of subsidized services. The government should exclude wealthy individuals and offer the subsidy only to low-income residents. The limited budget provided by the subsidy program should be allocated to low-income elderly individuals with missing teeth who are in dire need of financial support to improve their access to denture restoration services.

## Supplementary Information

**Additional file 1.** Data sets

## Data Availability

The datasets supporting the method and materials of this article are included within the References section and the additional files.

## Abbreviation

RPD: Removable partial denture
