# Supplementary material for: Access to denture restoration services under removable dentures subsidy program for adults aged 65 years and older in Taiwan- an interpretive approach
Source: BMC Health Serv Res. 2022 Jan 20;22:90. doi: 10.1186/s12913-022-07504-6 (PMC8781473; doi:10.1186/s12913-022-07504-6)
Supplement: Supplementary file 1 — Additional file 1. Data sets [file 12913_2022_7504_MOESM1_ESM.pdf]

Data set:

https://dep.mohw.gov.tw/domhaoh/cp-486-39243-107.html
https://dep.mohw.gov.tw/domhaoh/cp-486-1917-107.html
https://ws.moi.gov.tw/001/Upload/OldFile/site_stuff/321/2/year/year.html
https://dep.mohw.gov.tw/dos/cp-4033-42732-113.html
https://dph.tycg.gov.tw/care/home.jsp?id=246&parentpath=0,241
